# Predictive scoring system for risk of complications in pediatric dengue infection

**DOI:** 10.12688/f1000research.111214.1

**Published:** 2022-04-21

**Authors:** Monisha Bhaskar, Soundarya Mahalingam, Harish M M, Basavaprabhu Achappa

**Affiliations:** 1 Department of Pediatrics, Kasturba Medical College, Mangalore, Manipal Academy of Higher Education, Manipal, India; 2 Department of Internal Medicine, Kasturba Medical College, Mangalore, Manipal Academy of Higher Education, Manipal, India

**Keywords:** Pediatric Dengue, Complications, Predictive scoring

## Abstract

**Background: **Dengue infection has been a worrisome cause of mortality and morbidity in children. Though numerous scoring systems have been developed, they are in the adult population or are too complicated for use in children. Pediatric dengue infection has a wide spectrum from a mild illness to severe complications and an unpredictable course. Hence the need for a predictive scoring system where the possibility of complications can be identified which can contribute to reduction in mortality and morbidity of dengue by prompt referrals and anticipatory management.

**Methods:** Prospective case cohort study of children with confirmed dengue fever.

**Results**: 303 children were included and divided into two groups – the dengue fever group and the complicated dengue group based on the WHO clinical classification. The clinical and laboratory parameters were analysed individually, cut offs identified by ROC curves and compared for significance between the two groups. The parameters that emerged were hypotension, PCV ≥ 42%, platelet count ≤ 75000 cells/cumm, WBC ≥ 7000 cells/cumm, and ALT ≥ 70U/L.  Using the adjusted odd’s Ratio, and coefficient, individual predictive scores were tabulated ranging from 0 to 3, with a total score of 0 to 7. A cut-off score of 2 was then identified based upon the sensitivity (84.13%) and specificity (72.50%) as the ideal score to predict complicated dengue. Internal validation of the score was done where the area under the curve for predicting complicated dengue was 0.86 (95% CI 0.8-0.92) with a P value of <0.001. **Conclusion**: Our dengue predictive scoring system has been developed using five indicators, with a score of two and above, out of seven, suggesting increased risk of developing complications. This has been validated internally and can be used to predict complicated dengue among children.

## Introduction

Dengue fever is a serious public health issue that has resulted in significant mortality and morbidity in both children and adults. The environmental conditions in India, especially South India, play a vital role in the transmission of the infection with multiplication of the vectors, hence the monsoon seasons from June to September here see a surge in the dengue cases. Natural reservoirs of clean water like lakes, back waters as well as artificial water collections like the stagnant water in paddy fields, coconut shells collecting rainwater, village ponds, etc provide fertile grounds for the breeding of the Aedes mosquito.

Dengue infection has a myriad of clinical presentations which range from simple viral fevers with myalgia to complicated dengue with Dengue Shock Syndrome (DSS), Dengue Haemorrhagic Fever (DHF), Multiorgan dysfunction and Death. Each year the morbidity of dengue virus has been increasing with statistics from the NVBDCP 2013 (National Vector Borne Disease Control Program) showing that Kerala reported the highest prevalence of dengue (7911) with Karnataka becoming one of the endemic states with an incidence of 6048 cases out of 74454 cases all over India. ^1^ This incidence has only been increasing since then to 1,11,880 cases with 227 deaths in 2016. ^2^ This increasing incidence has reached 188401 cases with 325 deaths in 2017 and in 2018 – 101192 cases with 172 deaths, 2019 – 157315 cases with 166 deaths, 2020 – 39419 cases with 56 deaths all over India. ^3^^,^^4^

Dengue fever takes a severe toll among high-risk populations like children and elderly and is linked to an increased risk of morbidity, complications, and death. Studies on dengue fever in children have also reported worse outcomes both with respect to complications as well as death in paediatric dengue with an increased incidence of complications among adolescents. ^4^ The clinical features of dengue infection can extend from mild uncomplicated dengue infection with fever, rash, myalgia and arthralgia to gradually worsening complicated dengue. Severe complicated dengue infection implies intractable hypotension and shock, massive bleeding, capillary leak with accumulation of fluid in the third spaces like pleura, peritoneum and pericardium, multiorgan dysfunction due to inflammation causing liver dysfunction, acute kidney injury, cardiac failure following myocardial dysfunction, encephalitis and other neurological complications and eventual death. These complications are worse when the dengue infection affects the same child for the second time implying the extensive activation of the immune system when the infection occurs for the second time causing severe complications. ^5^ In line with this, adolescents have a worse course of the disease as probably they have been exposed to the infection in a mild form earlier in childhood. ^6^ The investigations done to identify dengue infection are the NS1 antigen which is done in the first few days of the illness and the IgM Dengue test which identifies the antibody response to dengue infection after the first 5 days of infection. Other tests are for organ dysfunction which is known to occur with complicated dengue infection which are done regularly as biochemical deterioration precedes clinical deterioration. Treatment in dengue infection is only supportive as there is no cure. It involves administration of iv fluids, vasopressors to increase the blood pressure and other care like mechanical ventilation, dialysis, etc based on the complications that develop. The more the complications that develop, the poorer is the outcome of the disease. ^7^ In children especially the management of fluids and the critical care are a challenge with the rapid progression of the disease as well as the delicate physiological balance in children. In view of this, early identification and watchful expectancy of complications are the only ways to anticipate worsening clinical condition in paediatric dengue.

It is with these considerations that the present study was conducted to analyse the parameters of pediatric dengue infection and develop a predictive scoring system based upon the laboratory and clinical parameters for the prediction of risks of complications in paediatric dengue.

## Methods

### Study design

This was a prospective case cohort study.

### Study population

All children aged 0 – 18 years with dengue infection.

### Study duration

Five years, from Jan 2016 to December 2020.

### Study setting

Hospital based study in the two tertiary medical college hospitals associated with our medical college.

*Sample size*: 270 (Calculated based on a previous study by Pongpan *et al* ^8^)

$$n=Z^{2}{\mathrm{pqd}}^{2}$$

where n = sample size

n = (1.96) ^2^ (0.51) (1-0.51) p = proportion of interest (0.06) ^2^, q = 1-p and d = relative precision.

In order to cover the time period, all cases in this time interval were included with a minimum of 270. Hence all 303 cases were considered.

### Inclusion criteria

All cases of confirmed dengue cases with NS1 antigen positivity confirmed by IgM dengue ELISA positivity after 5 days.

### Exclusion criteria

All cases who had an acute febrile illness with clinical features of dengue fever but negative diagnostic tests.

The study was submitted to the ethics committee of the institution (Institutional Ethics Committee, Kasturba Medical College, Mangalore. Ref No ECR/541/Inst/KA/2014/RR – 17) and clearance was obtained. The IEC certification was obtained before initiation of the study (IEC KMCMLR 10-16/497). Informed and written consent of all parents of the children and assent for adolescents was obtained before their inclusion into the study.

Demographic details, mode of presentation, clinical findings, laboratory parameters, treatment parameters and outcomes of all the included children were recorded and taken up for further analysis. The children were classified into two groups – The dengue fever group and the complicated dengue group based upon the WHO classification.

### Statistical analysis

The following steps were used:

1. Data were entered into Microsoft Excel, and statistical analysis was performed using SPSS 17.0 software. Qualitative variables were reported as frequency and percentages. Depending on the distribution of data, quantitative variables were reported as mean (standard deviation) or median (range).
2. The variables included in the study under demographic details, clinical features, examination findings with laboratory parameters were compared between the 2 groups i.e., Dengue fever group and complicated dengue group using Chi-square test (Bivariate analysis). Individual ROC curves were generated for the variables and cut off values were identified.
3. All the parameters were then compared for statistical significance between the groups using a multivariable regression model by binary logistic regression. This is was done in two steps in order to avoid loss of significance among parameters, hence the first step of the binary logistics was done with a p value less than 0.2. Among the parameters that emerged the next step was to identify statistical significance with the variables that showed statistical significance where p < 0.05. The effect measure was determined using an adjusted odds ratio with a 95% confidence interval.
4. The variables which were significant in the logistic regression model (p<0.05) were used to develop a score. The smallest coefficient from the model was given a score of 1 and scores for other significant variables were derived hence by dividing them by the smallest coefficient and the scores were rounded off to nearest 0.5.
5. The scores were applied for the variables and the total score was calculated by summing the scores. The area under the curve (AUC) with a 95% confidence interval was computed using a ROC curve. When an AUC>0.5, then the test applied is considered as a useful test. The Youden Index (Sensitivity+specificity-1) was computed and a cut-off for the score was chosen. Sensitivity, specificity, and predictive values were calculated with 95% CI.

## Results

A total of 303 children with pediatric dengue infection were analysed. They were divided into two groups - dengue fever group (240) and complicated dengue group (63) based upon the WHO criteria. A total of two demographic, ten clinical indicators and eight laboratory indicators were computed for each patient. ROC was generated for these individual parameters that were tabulated and cut off points were identified as shown in Table 1.

**Table 1.** Patient profiles in dengue fever and complicated dengue groups.

Patient profiles and cut off by ROC		Dengue fever (n=240) f (%)	Complicated dengue (n=63) (%)	P value
Demographic details				
Age ≤6y		50(83.3%)	10(16.7%)	0.379
Age >6y		190(78.2%)	53(21.8%)	
Gender: Male		131(81.4%)	30(18.6%)	0.324
Gender: Female		109(76.8%)	33(23.2%)	
Clinical indicators				
Duration of fever	<6 days	193(82.1%)	42(17.9%)	0.020
	≥6 days	47(69.1%)	21(30.9%)	
Headache	Present	68(73.9%)	24(26.1%)	0.134
	Absent	172(81.5%)	39(18.5%)	
Myalgia	Present	71(78.0%)	20(22.0%)	0.739
	Absent	169(79.7%)	43(20.3%)	
Abdominal pain	Present	36(72.0%)	14(28.0%)	0.169
	Absent	204(80.6%)	49(19.4%)	
Rash	Present	12(75.0%)	4(25.0%)	0.670
	Absent	228(79.4%)	59(20.6%)	
Vomiting	Present	92(72.4%)	35(27.6%)	0.014
	Absent	148(84.1%)	28(15.9%)	
Breathlessness	Present	1(50.0%)	1(50.0%)	0.307
	Absent	239(79.4%)	62(20.6%)	
Bleeding manifestations	Present	1(16.7%)	5(83.3%)	<0.001
	Absent	239(80.5%)	58(19.5%)	
Hypotension	Present	9(23.7%)	29(76.3%)	<0.001
	Absent	231(87.2%)	34(12.8%)	
Organomegaly	Present	2(22.2%)	7(77.8%)	<0.001
	Absent	238(81.0%)	56(19.0%)	
Laboratory parameters				
Haemoglobin (gm/dl)	<13	116(84.1%)	22(15.9%)	0.05
	≥13	124(75.2%)	41(24.8%)	
Packed cell volume (PCV) (%)	<42	189(84.4%)	35(15.6%)	<0.001
	≥42	51(64.6%)	28(35.4%)	
Lowest WBC (cells/cumm)	>3200	128(76.6%)	39(23.4%)	0.223
	≤3200	112(82.4%)	24(17.6%)	
Platelet count (cells/cumm)	>75000	163(89.6%)	19(10.4%)	<0.001
	≤75000	77(63.6%)	44(36.4%)	
Alanine aminotransferase (U/L)	<70	205(86.9%)	31(13.1%)	<0.001
	≥70	35(52.2%)	32(47.8%)	
Higher WBC (cells/cumm)	<7000	192(84.2%)	36(15.8%)	<0.001
	≥7000	48(64.0%)	27(36.0%)	
Creatinine (mg/dl)	<0.5	133(79.6%)	34(20.4%)	0.837
	≥0.5	107(78.7%)	29(21.3%)	
Ferritin (n=191) (ng/ml)	<1000	89(86.4%)	14(13.6%)	<0.001
	≥1000	45(51.1%)	43(48.9%)	
Ferritin (n=191) (ng/ml)	<1500	103(83.1%)	21(16.9%)	<0.001
	≥1500	31(46.3%)	36(53.7%)	

Following this, Multivariate regression analysis was done by binary logistics ( Table 2). As an effect metric, the adjusted odds ratio with a 95% confidence interval was determined. Clinical & laboratory characteristics with substantial prediction potential for dengue severity were identified in the multivariable analysis as Hypotension as the strongest predictor with an Odd’s ratio of 20.11, Packed cell volume (>42%), Platelet count (<75,000/cumm), WBC count (>7000/cumm) and ALT (>70 IU/ml). Ferritin was not included in multivariable regression analysis because of missing values.

**Table 2.** Binary logistic regression of variables with P value <0.2.

Variable	Category	Coefficient	Adjusted OR	95% CI		P value
				Lower	Upper	
Duration of fever	≥6 days	0.23	1.25	0.53	2.97	0.606
Vomiting	Yes	0.32	1.38	0.63	3.02	0.417
Abdominal pain	Yes	0.13	1.14	0.42	3.06	0.796
Bleeding manifestations	Yes	1.79	6.00	0.48	75.58	0.165
**Hypotension**	**Yes**	**3.0**	**20.11**	**7.50**	**53.89**	**<0.001**
Organomegaly	Yes	1.49	4.44	0.61	32.41	0.141
Haemoglobin (gm/dl)	≥13	0.02	1.02	0.40	2.60	0.970
**PCV (%)**	**≥42**	**0.94**	**2.56**	**1.04**	**6.33**	**0.041**
**Platelet count (cells/cumm)**	**≤75000**	**1.06**	**2.89**	**1.30**	**6.39**	**0.009**
**WBC (cells/cumm)**	**≥7000**	**1.16**	**3.19**	**1.39**	**7.33**	**0.006**
**ALT (U/l)**	**≥70**	**1.07**	**2.92**	**1.24**	**6.90**	**0.015**

**Figure 1.**
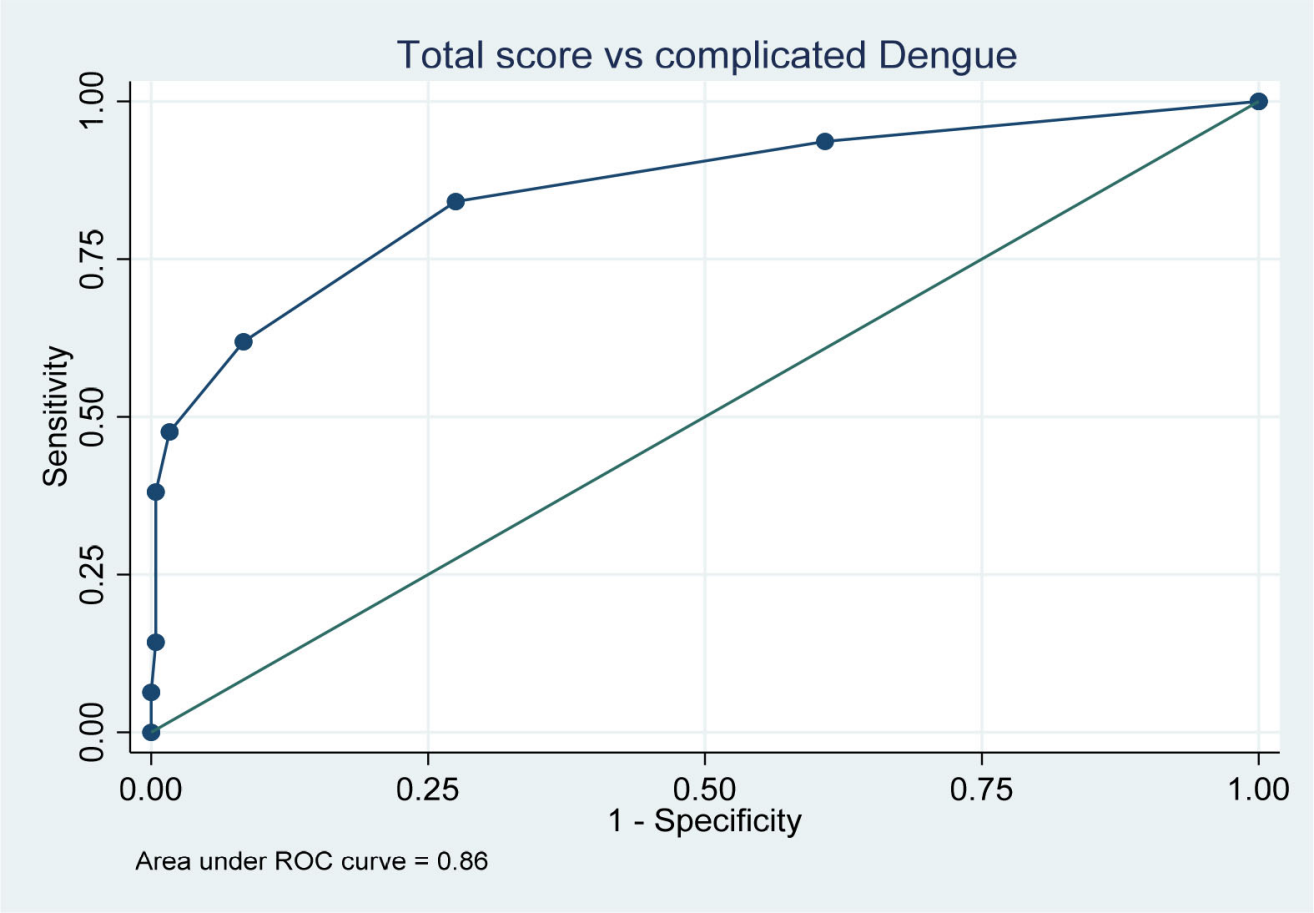
Area under the curve for the calculated predictive scoring.

Among the parameters that emerged statistically significant, coefficients were calculated with the adjusted Odd’s Ratio and then analysed to develop a score ( Table 3). Significant parameter coefficients were converted into item scores by dividing each coefficient by the model's smallest coefficient (0.94) and rounding up or down to the nearest 0.5 integers. Individual predictive scores ranged from 0 to 3, with a total score of 0 to 7 ( Table 3). To check the usefulness of the test and score, area under the curve was plotted against sensitivity and 1 – specificity. The area under the curve was calculated as 0.86 with a 95 percent confidence interval (CI) of 0.8-0.92, showing statistical significance with a p value of < 0.001, hence making this scoring a useful predictive test ( Figure 1).

**Table 3.** Significant predictors of complicated dengue.

Selected Variable	Category	Adjusted OR	95% CI		P Value	Coefficient	SCORE
			Lower	Upper			
**PCV (%)**	<42	1					0
	≥42	2.56	1.04	6.33	**0.041**	0.94	**1**
**Platelet count (cells/cumm)**	>75000	1					0
	≤75000	2.89	1.30	6.39	**0.009**	1.06	**1**
**WBC (cells/cumm)**	<7000	1					0
	≥7000	3.19	1.39	7.33	**0.006**	1.16	**1**
**ALT (U/L)**	<70	1					0
	≥70	2.92	1.24	6.90	**0.015**	1.07	**1**
**Hypotension**	No	1					0
	Yes	20.11	7.50	53.89	**<0.001**	3.0	**3**

The sensitivity and specificity of the score was calculated at different cut off points and their individual Youden index (Sensitivty+specificity-1) was calculated ( Table 4), to identify the ideal score where the sensitivity and specificity would be appropriate. The highest index was found at a score of two (out of seven) with a sensitivity of 84% and a specificity of 72.5%. This was the score chosen so as to not overlook the complications early at admission in pediatric dengue which can progress fast into complications if undetected early.

**Table 4.** Sensitivity and specificity of scores at different cut -off points and Youden index.

Score cut off	Sensitivity	Specificity	Correctly classified	Youden index
(≥0)	100.00%	0.00%	20.79%	0.00
(≥1)	93.65%	39.17%	50.50%	0.33
**(≥2)**	**84.13%**	**72.50%**	**74.92%**	**0.57**
(≥3)	61.90%	91.67%	85.48%	0.54
(≥4)	47.62%	98.33%	87.79%	0.46
(≥5)	38.10%	99.58%	86.80%	0.38
(≥6)	14.29%	99.58%	81.85%	0.14
(≥7)	6.35%	100.00%	80.53%	0.06
(>7)	0.00%	100.00%	79.21%	0.00

**Figure 2.**
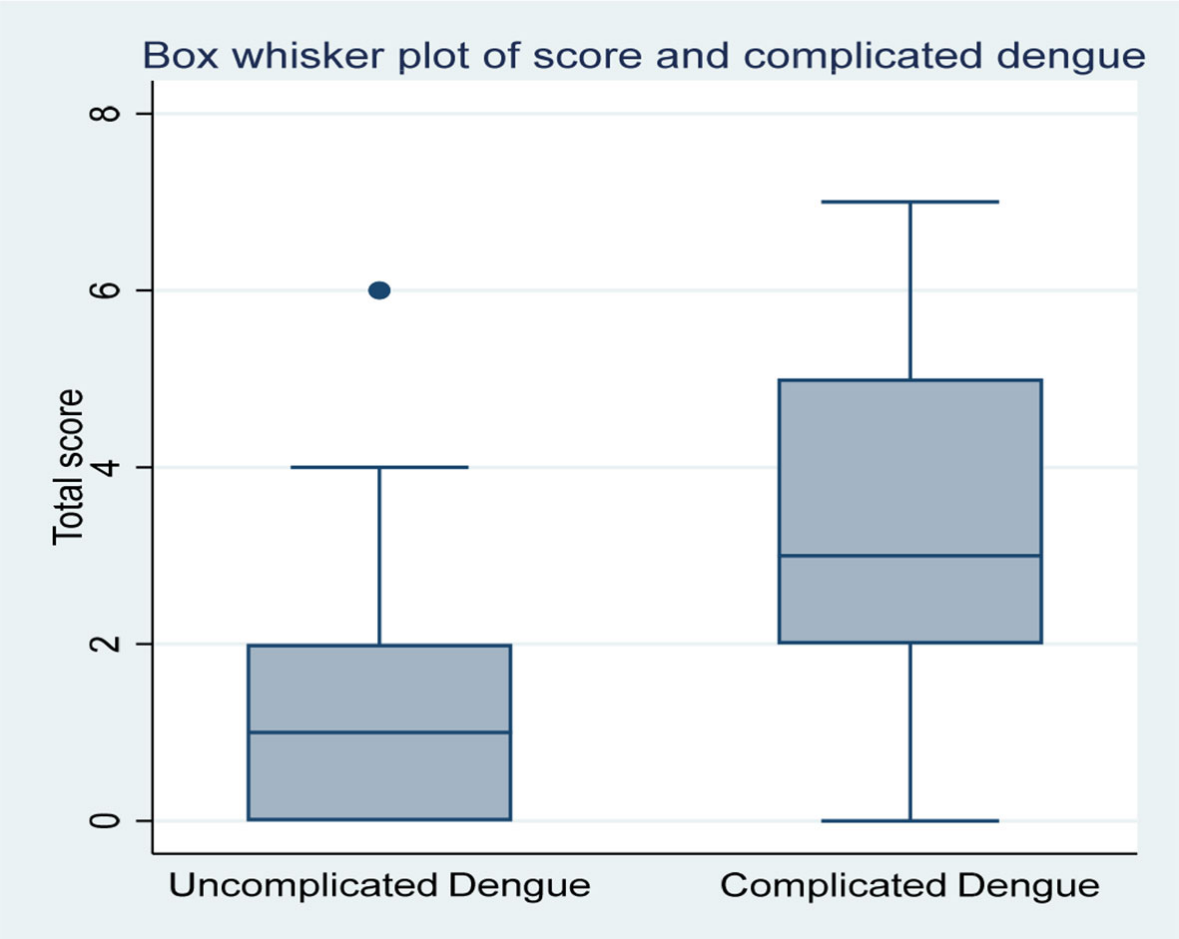
Distribution of Dengue Severity Scores using the predictive model in the sample population.

Our score was then applied to our data and the patients were split into two groups: Scores <2 (dengue fever) and scores ≥2 (complicated dengue) as shown in Figure 2. The scores <2 predicted dengue fever correctly in 174 out of 240 patients and the scores ≥2 predicted complicated dengue correctly in 53 out of 63 patients. The sensitivity of our dengue predictive scoring method is 84.1%, the specificity is 72.5%, the positive predictive value is 44.5 % and the negative predictive value is 94.5%. When the mortality statistics were analysed, there were 4 deaths in the cohort. When the score was applied, all the four deaths had a score of >2.

## Discussion

The complications and unpredictable outcomes of dengue infection in a child necessitates the requirement for early identification and anticipatory management. As we reviewed literature, few scoring systems were available for the prediction of complications in dengue. Most of these scoring systems were in adults and could not be applied to children.

Generalised scoring systems like the Pediatric Risk of Mortality III (PRISM III) and Pediatric Logistic Organ Dysfunction (PELOD) scores showed good discrimination in predicting mortality and complications when children with dengue are admitted to the PICU. ^9^ However, in such generalised systems, the outcomes of the PICU admission are validated better and these scores require numerous investigations including arterial blood gas analysis. They are not specific to dengue, nor help in early and clinical prediction of development of complications. Dengue infections were also classified as DF, DHF grade I, II and III, with decision tree algorithms which were also used to determine whether or not hospitalisation was required. ^10^^,^^11^ These algorithms were more applicable in adults and not specific for pediatric dengue. The algorithm by Lee *et al* ^11^ shows data mining of the dengue infection by decision tree and this uses numerous calculations and has shown application in engineering mathematics as it is difficult to apply clinically. Scores by Phakhounthong K *et al* ^12^ showed the development of the score using classification tree analysis, here the algorithm uses the Glasgow Coma Score, hematocrit, creatinine, platelet count and urine protein. This score relies on the clinical judgement of the pediatric GCS which is difficult in smaller children as well as gives the value of S.creatinine which is abnormal as 0.95 mg/dl. These lab parameters are not available immediately and the golden hour at admission gets missed. Hence in our score, we have focussed upon clinical parameters like the vital signs at admission as well as the lab parameters which are easily available even in smaller centres and laboratories. Another scoring system was developed by Huang ^13^ which was to predict mortality in dengue fever but was in adults and as age with comorbidities were significant parameters, this cannot be used in children. All these scoring systems/algorithms ^11^^,^^13^^,^^14^ had limitations, for example, the study population didn’t include pediatric cases, no specific clinical or laboratory parameters were included related to dengue infection nor such scoring systems were developed in our geographical setting.

The closest scoring system that we could find in literature was given by Pongpan *et al* ^8^ here in 198 children in Thailand, they developed a score with six parameters (Age, hepatomegaly, hematocrit, systolic BP, WBC count and platelet counts) with a total score of 18, however in this scoring, clinical judgement of hepatomegaly had the highest weightage of 8.5/18 and this judgement decides the score of the child. Such clinical parameters are not easily applied in a peripheral setting where pediatric examination is difficult by basic medical officers thus making both overdiagnosis and underdiagnosis a problem. Another significant aspect of the score by Pongpan *et al* was that Systolic BP with a cut off of 90 mmHg was selected. The drawback of this is that, in smaller children where the 50th centile of BP is around 90 mmHg, this score will over diagnose positive markers of complications even if the BP is normal as for age. In our score Hypotension has been given a priority where the detection of hypotension was by taking age dependent cut offs which is easily done even in peripheral centres.

Hence to summarise, in consideration of all the above, we created a dengue severity scoring system that uses laboratory indicators and clinical signs to predict the risk of complications and fatality in pediatric dengue infection using binary logistic regression. The final scoring system for predicting complicated dengue comprised five components: PCV, Platelet count, ALT, Highest WBC and Hypotension. All these parameters are easily identified in any setting, tertiary or peripheral. The applicability of the score is easier as well as more relevant. Hypotension was defined by age dependent charts and was found to be the most important predictor of complicated dengue. The laboratory predictors included in our scoring system are simple to perform in outpatient hospital labs, and the scoring method is simple to apply in daily clinical practice. While applying the score, patients with a severity score of zero or one can be treated in the outpatient setting. A patient with a severity score of two may require hospitalisation for close observation. Admission to an intensive care unit, early resuscitation, empiric antibiotics, and other supportive measures are required if a patient's severity score is three or higher.

One of our study's limitations is the small sample size of the study population as larger samples would give better opportunities to internally validate our scoring system.

## Conclusions

The significant predictors of complicated dengue were Hypotension, PCV ≥ 42%, Platelet count ≤ 75000 cells/cumm, WBC ≥ 7000 cells/cumm, and ALT ≥ 70U/L. Individual predictive scores ranged from zero to three, with a total score of zero to seven. Score of ≥ two indicates patients going in for dengue related complications. The sensitivity of our dengue predictive scoring method is 84.1%, the specificity is 72.5%, the positive predictive value is 44.5% and the negative predictive value is 94.5%. The dengue prediction score system includes five clinical and laboratory markers that can be used to predict complicated dengue in paediatric patients. Validation of our predictive scoring system is needed before its application in routine clinical practice.

## Data availability

### Underlying data

Dryad: Mahalingam, Soundarya; Bhaskar, Monisha; Achappa, Basavaprabhu, MM Harish (2022), Predictive Scoring for Risk of Complications in Pediatric Dengue infection, Dryad, Dataset.

DOI: https://doi.org/10.5061/dryad.wpzgmsbpp

Link for data set:

https://datadryad.org/stash/share/znfJ64hSeNFEcrPs-PUpcu8VIDOSkEiPmrskbMtagl8.

This project contains the following underlying data:

• Data file 1. (Excel spreadsheet of all cases)
• Data file 2. (Readme file for coding of the spreadsheet)

Data are available under the terms of the Creative Commons Zero “No rights reserved” data waiver (CC0 1.0 Public domain dedication).
