# Supplementary material for: Predictive scoring system for risk of complications in pediatric dengue infection
Source: F1000Res. 2022 Apr 21;11:446. [Version 1] doi: 10.12688/f1000research.111214.1 (PMC9263579; doi:10.12688/f1000research.111214.1)
Supplement: Peer review history

# Peer review: Reviewer response for version 1

**DOI:** 10.5256/f1000research.122909.r135433

**Published:** 2022-07-07

**Authors:** Farhan Fazal

**Affiliations:** 1 Department of Infectious Disease, Sheikh Khalifa Medical City, Abu Dhabi, United Arab Emirates

This article is on the Development of a predictive scoring system for dengue infection among children so as to detect complications early. Thank you for giving me the opportunity to review this article. In the article, the authors have reviewed cases and developed a score that can applied at the point of care to identify the early development of complications.

1. One of the important aspects of the score is that it relies primarily on clinical parameter of hypotension along with basic laboratory parameters that are available in a peripheral lab as well.
2. The statistical methods have been done well and explained in much detail for the ease of understanding. The steps outlined can be made concise in a flowchart as well. The Youden index and the multivariate regression is appropriately done as required for such a predictive scoring development.
3. A good aspect of this study is that the authors have done an internal validation of their scoring with their study sample and have shown that the scoring is appropriate.
4. The discussion has compared many scoring systems that have been developed along with the pros and cons of such scoring systems. The study is done on children and there are few studies that are available for comparison which has been done by the authors. Adult scoring systems have also been detailed and their difficulties have been listed out. Hence this is a relevant scoring system for use in children with dengue.

Some clarifications required are:

1. Details of the serum ferritin as to why two cut offs of 100mg/dl and 1500mg/dl were used needs to be addressed in the methodology and results

Is the work clearly and accurately presented and does it cite the current literature?

Yes

If applicable, is the statistical analysis and its interpretation appropriate?

Yes

Are all the source data underlying the results available to ensure full reproducibility?

Yes

Is the study design appropriate and is the work technically sound?

Yes

Are the conclusions drawn adequately supported by the results?

Yes

Are sufficient details of methods and analysis provided to allow replication by others?

Yes

Reviewer Expertise:

Infectious disease

I confirm that I have read this submission and believe that I have an appropriate level of expertise to confirm that it is of an acceptable scientific standard.

# Peer review: Reviewer response for version 1

**DOI:** 10.5256/f1000research.122909.r135434

**Published:** 2022-05-04

**Authors:** Sanjay Kumar Sahu, Asish Ranjan Mohakud

**Affiliations:** 1 Pediatrics, Kalinga Institute of Medical Sciences, Bhubaneswar, India; 2 Pediatric Cardiology, Kalinga Institute of Medical Sciences, Bhubaneswar, India

Thanks for giving us the opportunity to review this article. It is an excellent article clearly and accurately presented and cites the current literature.

 However there are few points that needs clarification:

1. Regarding inclusion criteria you have included all confirmed dengue cases with NS1 antigen positivity confirmed by IgM dengue ELISA positivity after 5 days. But what about those cases that are IgM negative after 5 days with previous NS1 positivity?
2. Regarding the cut-off value of serum creatinine (Table 1) in a sample of children aged 0 to 16 years, a single cut-off value of 0.5mg/dl may not be appropriate for all. This can be adjusted to BSA for uniform comparison.  
3. We are unable to understand the basis of taking 2 cut-off values for serum ferritin of 1000ng/ml and 1500ng/, as greater than 1000ng/ml was statistically significant in your analysis in Table 1. The 2 cut-off values of serum ferritin can be adjusted to a single value.

Is the work clearly and accurately presented and does it cite the current literature?

Yes

If applicable, is the statistical analysis and its interpretation appropriate?

Yes

Are all the source data underlying the results available to ensure full reproducibility?

Yes

Is the study design appropriate and is the work technically sound?

Yes

Are the conclusions drawn adequately supported by the results?

Yes

Are sufficient details of methods and analysis provided to allow replication by others?

Yes

Reviewer Expertise:

Pediatrics infectious disease, Pediatrics Asthma, Allergy & Immunology

We confirm that we have read this submission and believe that we have an appropriate level of expertise to confirm that it is of an acceptable scientific standard.
